# Toll-Like Receptors: Regulators of the Immune Response in the Human Gut

**DOI:** 10.3390/nu10020203

**Published:** 2018-02-13

**Authors:** Hubert Hug, M. Hasan Mohajeri, Giorgio La Fata

**Affiliations:** 1 DSM Nutritional Products Ltd., R & D Human Nutrition and Health, P.O. Box 2676, 4002 Basel, Switzerland; hubert.hug@dsm.com (H.H.); hasan.mohajeri@dsm.com (M.H.M.); 2 University of Zurich, Winterthurerstrasse 190, 8057 Zürich, Switzerland

**Keywords:** TLR2, TLR4, TLR5, immune system, gastrointestinal tract, bacterial ligands

## Abstract

Toll-like receptors (TLRs) are powerful molecular regulators by which the immune system may “sense” the environment and protect the host from pathogens or endogenous threats. In mammalian cells, several TLRs were identified with a tissue and cell type-specific distribution. Understanding the functions of specific TLRs is crucial for the development and discovery of compounds useful to maintaining or re-establishing homeostasis in the gastrointestinal tract (GIT). Due to their relevance in regulating the inflammatory response in the GIT, we will focus here on TLR2, TLR4, and TLR5. In particular, we describe (a) the molecular pathways activated by the stimulation of these receptors with their known bacterial ligands; (b) the non-bacterial ligands known to interact directly with TLR2 and TLR4 and their soluble forms. The scope of this minireview is to highlight the importance of bacterial and non-bacterial compounds in affecting the gut immune functions via the activation of the TLRs.

## 1. Introduction

Being in proximity to the largest interface between the body and the external environment, the gut immune system has evolved to distinguish the tiniest molecular difference that exists between pathogenic and non-pathogenic microorganisms. It has developed the capacity to react rapidly to any harmful threats before the “enemy” can multiply or even before harmless bacteria may mutate into dangerous ones [1,2]. Therefore, in the gastrointestinal tract (GIT), the immune system contributes to the maintenance of the delicate equilibrium that exists between bacteria, yeast, bacteriophages, and gut epithelium to guarantee homeostasis and the optimal health of the organism.

**Figure 1:**
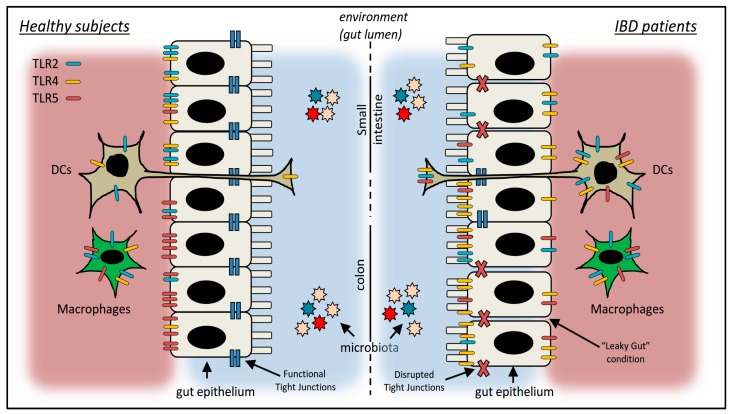
Schematic representation highlighting the expression of Toll-like receptors (TLRs) in the gastrointestinal tract (GIT) of healthy subjects and patients with inflammatory bowel disease (IBD). The gut epithelium represents the interface between the microbial ecosystems present in the lumen of the GIT and the immune system. In healthy conditions, the barrier function of the gut epithelium is guaranteed by the tight junctions between the cells. In pathological conditions, these junctions are disrupted, exposing their basolateral receptors to potentially pathogenic ligands (“Leaky Gut” condition). TLR2, TLR4, and TLR5 are expressed on the gut epithelium cells, as well as on cells of the immune system (here represented only by macrophages and dendritic cells (DCs)). The localization of the specific TLRs is also indicated in the small intestine and colon specifically. Details and references are reported in the text.

The immune reaction caused by an invading pathogen needs to be properly tackled, avoiding underestimations, but also without developing over-reactions deleterious for the host organism. Hence, the first signals the pathogen-sensing cells recognize are the most crucial ones. An early interaction point occurs between potentially pathogenic bacteria and the toll-like receptors (TLRs) present on the membrane of the gut immune cells, as well as on the cellular membrane of the gut epithelium [3,4] (Figure 1). In some cases, soluble forms of the TLRs also exist [5,6] that have a role in limiting an overstimulation of the immune system.

TLRs belong to the pattern recognition receptors (PRRs) family. They recognize molecules broadly shared by pathogens collectively named as pathogen-associated molecular patterns (PAMPs) and microbe-associated molecular patterns (MAMPS), the latest usually expressed by resident microbiota [7,8,9,10]. Moreover, TLRs recognize endogenous molecules derived by tissue damage and accordingly defined as damage-associated molecular pattern molecules (DAMPs) [7,11,12,13]. The majority of the endogenous ligands are extracellular matrix molecules such as fibronectin, heparan sulphate, biglycan, fibrinogen, oligosaccharides of hyaluronan, and hyaluronan breakdown fragments [14,15,16,17,18,19,20,21]. In physiological conditions they are considered inert because, due to a different compartmentalization, they are uncapable of interacting with their receptors. On the contrary, in pathological conditions, endogenous ligands are passively and actively released by the cells and, interacting with specific TLRs (TLR2 and TLR4 in particular), they can regulate the inflammatory response in the tissue [21].

Therefore, TLRs on one hand sense the potentially harmful molecules of pathogens that infect humans [6] and switch on the body’s defense systems to react accordingly, i.e., by activation of the innate immune system first, and later—with help of other cells—by activation of the adaptive immune response [22]. On the other hand, they may inhibit the inflammatory cascade that could be triggered by the continuous interaction between host and commensal bacteria.

Upon binding with the pathogenic ligands, TLRs form homodimers or heterodimers, and initiate signaling cascades leading to the release of pro or anti-inflammatory cytokines or antiviral compounds (i.e., type I interferon (IFN)) [23].

In this minireview, we focus on TLR2, TLR4, and TLR5, since among all TLRs, these are the main extracellular receptors that interact with the microbiota in the human GIT. We will describe the ligands responsible for their activation and the induced signaling pathways. A specific section is also dedicated to the description of plant-derived compounds that may regulate the inflammatory response in the GIT via TLR activation. Understanding the dynamic equilibrium that exists between microorganisms in the gut and the activity of the immune system regulated by TLRs is crucial for the development and discovery of compounds critical to maintaining or re-establishing homeostasis in the GIT such as pre- and probiotics, as well as pharma- and nutraceuticals.

## 2. Expression of TLRs in the Gut

Consistent with their roles in immune surveillance, TLRs expression is elevated in those tissues exposed to external environments such as lung and GIT [24] with patterns that are unique for specific cell types [25]. In mammals, 13 different TLRs have been identified (human: TLR1-11; mouse: TLR1-9, TLR11-13) with few functional differences observed between humans and mice [26].

Generally, TLRs that respond to bacterial products such as triacyl and diacyl lipoproteins, lipopolysaccharide (LPS), and flagellin are transmembrane receptors expressed on the cellular membranes of the immune and gut epithelium cells. TLR2, together with its heterodimer partners TLR1 and TLR6, and TLR5, belong to this category. By contrast, nucleic acid-sensing TLRs such as TLR3, TLR7, TLR8, TLR9, TLR11, TLR12, and TLR13 are exclusively expressed on the membrane of intracellular compartments. TLR4 instead, is expressed on the cellular membranes (pro-inflammatory signaling), as well as intracellularly (anti-inflammatory signaling) [26,27,28].

Using a combination of immunohistochemistry, enzymatic separation, and laser capture microdissection of the intestinal epithelium, it was shown that healthy human colon tissues contain lower levels of TLR 2, TLR4 in the intestinal epithelial cells (IECs), with higher levels of TLR5 [29,30]. TLRs expression by the IECs also shows a polarized pattern [25,29]. TLR2 and TLR4 are localized mainly in the basolateral surfaces of the enterocytes of the small intestine, while TLR5 is localized mainly on the basolateral surfaces of the enterocytes in the colon [25,29,31]. This compartmentalization pattern is key to ensuring that commensal bacteria do not induce an inflammatory response by the organism, and therefore it is crucial for the onset and progression of pathological conditions [25].

The expression pattern of specific TLRs extends also to other cell types of the intestinal epithelium such as Paneth cells, expressing TLR2, TLR4, and TLR5 [32], and cells of the immune system such as macrophages and dendritic cells (DCs) (Figure 1). Differently from the DCs present in the blood, only a small proportion of intestinal DCs express TLR2 and TLR4, with no significant differences between the DCs contained in the ileal and colonic tissues [33]. Macrophages also express TLR2 and TLR4, although at low levels in normal physiological conditions.

A different expression pattern for TLRs occurs in pathological conditions (Figure 1). Colonic IECs from patients with inflammatory bowel disease (IBD) have higher expression levels of TLR4 in particular [34], as well as lower levels of TLR2 and TLR5 [29]. The cellular localization of specific TLRs also changes in pathological conditions, as demonstrated in patients with active Crohn’s disease in which the basolateral expression of TLR4 in the colonic IECs is shifted to an apical position [29,30]. Differently from TLR4, the localization of TLR2 and TLR5 in the IECs remains unchanged when patients with active IBD are compared to healthy controls [30]. Of note, the expression levels of the TLR2 and TLR4 is increased in the inflammatory cells of the lamina propria [30] and specifically in the intestinal macrophages during inflammation [35]. The expression levels of TLR2 and TLR4 are also increased in the DCs of patients with IBD, with no differences between Crohn’s diseases and ulcerative colitis [33]. Finally, TLR2 and TLR4 expression is also increased in the intestinal macrophages contained in inflamed mucosa compared to healthy colon [35].

## 3. Signaling Pathways

**Figure 2:**
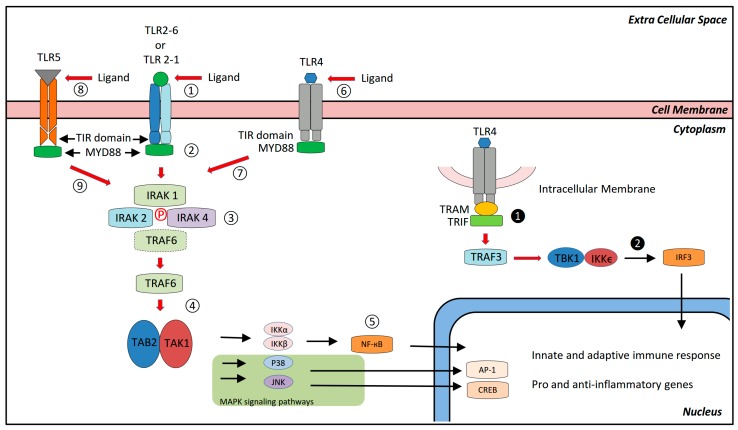
Schematic and simplified representation of the TLR2, TLR4, and TLR5 signaling pathways. Upon ligand recognition, TLR2 is activated and dimerizes with TLR1 or TLR6 ①. A MYD88-dependent intracellular pathway is therefore initiated with ligation of MYD88 (myeloid differentiation primary response protein 88) to the cytoplasmic TLR domain TIR (Toll-IL-1 receptor) ②. MYD88 recruits the IRAK (IL-1R-activating kinases) complex and TRAF6 (TNF-receptor associated factor 6). Phosphorylation of the IRAKs induce activation and release of TRAF6 from the complex ③. TRAF6 then activates the TAK1-binding protein 2 and TGF-activated kinase 1 (TAB2-TAK1) complex ④. Finally, the IκB kinases-α and β (IKKα and IKKβ) complex, responsible for activation of the transcription factor (NF-κB) that translocates into the nucleus, where it regulates gene expression of mainly pro-inflammatory genes ⑤. Moreover, TRAF6 activates the mitogen-activated protein kinases (MAPKs) signaling pathway (JNK and P38), whose final effect is the activation of transcription factors AP-1 and CREB. TLR4 homodimers, following TLR4-ligand interaction ⑥. Induce activation of a MYD88-dependent pathway ⑦, as well as MYD88-independent pathway ❶. The second pathway starts with recruitment of TRAM (TRIF-related adapter molecule) to the TIR domain of the TLR4 homodimers and follows with recruitment of TRIF (TIR domain-containing adaptor inducing IFN-β) ❶. TRIF activates TRAF3 and, finally, the transcription factor IRF3 (Interferon Regulatory Factor 3) via recruitment of TANK-binding kinase 1 (TBK1) and IKKϵ ❷. IRF3 translocates to the nucleus, where it induces the expression of type I interferon (INF) genes. TLR5 ligand (flagellin) activates TLR5 ⑧ and, because of this, the MYD88-dependent pathway described above and in the text ⑨. The final effect is the expression of pro-inflammatory genes.

Each TLR is composed of a leucine-rich ectodomain responsible for the recognition of the PAMPs, DAMPs, or MAMPs, a transmembrane domain, and a cytoplasmic Toll-IL-1 receptor (TIR) domain that initiate the signaling cascade [36,37,38]. The TIR domain may recruit (upon activation of the specific TLR) different adaptors that are afterwards responsible for a specific biological response. They are: MYD88, TRIF, TIRAP/MAL, and TRAM (see Figure 2 for details). MYD88 is utilized by all TLRs. TRIF is recruited to TLR3 and TLR4. TRAM is selectively recruited to TLR4 and mediates the association of TRIF-TLR4. TIRAP instead is a sorting adaptor that mediates recruitment of specific proteins to TLRs expressed in both cellular and intracellular membranes [36,39].

### 3.1. TLR2

TLR2 recognizes a wide range of ligands (see [40] for details), among which the best characterized are lipoproteins ubiquitously present in all bacteria and highly expressed on the outer membrane of gram-positive bacteria (Table 1). Accessory molecules and co-receptors have been described to concentrate microbial products in proximity of TLR2 and facilitate its activation [40]. An example is the Cluster of Differentiation 36 (CD36) that may bind specific ligands and transfer them to the accessory molecule CD14, which in turn loads the ligands onto TLR2-TLR1 and TLR2-TLR6 heterodimers [40].

Generally, activation of TLR2 induces binding of MYD88 followed by activation of transcription factors such as CREB, AP-1, and NF-κB that induce expression of pro-inflammatory cytokines (Figure 2 and [26] for details). Nevertheless, the capacity of TLR2 to induce pro or anti-inflammatory responses is associated with the different co-receptor it may dimerize with TLR1 or TLR6 [51,52]. Specifically, TLR2-TLR6 heterodimers activate the signaling cascade pathway responsible for transcription of pro-inflammatory molecules such as interleukin-6 (IL-6) and the tumor necrosis factor-alpha (TNF-α), while TLR2-TLR1 heterodimers induce an anti-inflammatory response via IL-10 expression [52,53].

IL-10 is a key molecule for gut homeostasis. On one side, IL-10 maintains the mucosal immune system in a partially unresponsive state to specific antigens [54,55] and controls the development of cells producing pro-inflammatory cytokines ([55,56], while on the other side it may dampen the MYD-88 pro-inflammatory signaling by ubiquitination and degradation of IRAK4 and TRAF6 ([55,57] and Figure 2). Moreover, the production of IL-10 is also stimulated by commensal bacteria (such as Bacteroides) that promote the development of IL-10-producing cells that contribute to the maintenance of health homeostasis [55,58]. For these reasons, loss of function mutations in the IL-10 receptor genes were recently associated with early onset of hyperinflammation in patients with IBD [59], as well as polymorphisms in the IL-10 gene demonstrated to be associated with ulcerative colitis [55,60]. Epidemiological data assessing the role of TLR2 in the pathogenesis of IBD are scarce; nevertheless, monocytes isolated from patients with active IBD had higher expression levels of TLR2 on their cell surfaces and a significantly increased TNF-α production in response to TLR2 agonist stimulation as compared to inactive patients and healthy controls [61,62].

Homodimerization of TLR2 was proposed but not yet observed [40,63], and, more recently, heterodimers of TLR2 and TLR10 were also described, although their function is unclear [40,64]. The heterodimer complexes not only provide a more specific signaling cascade, but also increase the variety of molecules (ligands) that TLRs can sense (due to the additional combinations). This suggests also that heterodimer complexes may exist already in the absence of ligands [40]. In addition, a recent study described interaction of TLR2 with TLR4 upon recognition of the HIV-1 gp120 protein [11,65], raising the hypothesis that other, yet unidentified, functions associated with specific heterodimers exist that could amplify the range of molecules recognized by TLRs.

### 3.2. TLR4

Several PAMPs including the lipopolysaccharide (LPS), as well as plants and viral motifs, can activate TLR4 [21,66,67]. LPS derives from the outer membrane of gram-negative bacteria, and it is one of the most studied immune-stimulatory components [68,69]. Upon LPS binding, TLR4 forms homodimers that induce the activation of two possible signaling pathways: MYD88-dependent and MYD88-independent (or TRIF-dependent) (Figure 2 and below for details). LPS-dependent activation of TLR4 strongly depends on the co-receptor CD14 that is specifically required for the detection of smooth LPS (abundant *O*-glycosylation) rather than rough LPS or lipid A [41]. Of note, TLR4 is expressed extracellularly and intracellularly in a fine balance between pro-inflammatory and anti-inflammatory (respectively) signaling roles [26].

TLR4 expression is upregulated in both Crohn’s disease and ulcerative colitis, and in pediatric IBD patients, higher levels of TLR4 mRNA and protein were found in the inflamed colonic mucosa when compared with non-inflamed controls [61,70]. Moreover, several epidemiological studies show an association between TLR4 polymorphism and susceptibility to IBD, although the data lack consistency when different cohorts are considered [61,71,72,73,74].

*MYD88-dependent pathway*: This pathway is the same as the one activated by TLR2 stimulation. Shortly, TLR4 homodimerization recruits MYD88 and the IRAK complex including TRAF6 that activates TAK1. Upon TAK1 and IKKβ activation, the transcription factor NF-κB translocates into the nucleus where, together with the MAPK activated AP-1, it regulates the expression of pro-inflammatory cytokines and related genes such as IL-6, TNF-α, IL-1, and others (Figure 2 and [69]).

*MYD88-independent pathway*: Upon activation of TLR4 by its ligand, TLR4 dimerizes and recruits TRIF via the adaptor protein TRAM. TRIF recruitment activates TRAF3 and subsequently the transcription factor IRF3, a member of the interferon regulatory transcription factor family, which becomes phosphorylated on serine/threonine. Phosphorylated IRF3, together with other proteins assembled in a complex, translocates into the nucleus and activates the transcription of type I INF genes (Figure 2) that mediate antiviral activity. In this way, IRF3 plays a crucial role in the activation of the innate immune system after viral infections [75].

### 3.3. TLR5

TLR5 recognizes flagellin, the key constituent protein of the bacterial flagellum present in all flagellated bacteria [50,76,77]. Upon flagellin ligation, TLR5 homodimerizes and activates the MYD88-dependent pathway described already for TLR2 and TLR4 (see Figure 2). TLR5 is, therefore, responsible for the induction of pro-inflammatory genes such as TNF-α, IL-6, IL-1β, etc. [78].

As already reported in Figure 1, TLR5 expression is mainly basolateral and confined to the colon epithelial cells where, in healthy conditions, it is not accessible to stimulation by its ligand: flagellin [61]. Nevertheless, under inflammatory conditions and, as a consequence of epithelial barrier disruption, TLR5 becomes accessible to flagellin and is therefore reactive to specific bacteria [61,78,79]. In Crohn’s disease patients, the tolerance to commensal-derived flagellin is lost, while an enhanced flagellin reactivity is gained [61,80,81]. Data connecting TLR5 polymorphism to IBD is limited. A point mutation in TLR5 was demonstrated to be responsible for a polymorphism associated with lower levels of IgG and IgA specific to flagellin in healthy individuals [61,82]. Of note, the carriage rate of the same polymorphism was significantly lower in Crohn’s disease patients when compared to their relatives or healthy individuals [61,83]. More recently, other TLR5 polymorphisms were described and associated with an increased risk for IBD in different ethnical cohorts [84,85].

### 3.4. Soluble TLRs

Some TLRs exist also in soluble forms (sTLRs) that function as decoy receptors to prevent a direct interaction between TLRs and their bacterial ligands [6]. Consequentially, sTLRs may limit an overstimulation of the immune system and prevent acute responses to infection [6]. sTLRs have been described for TLR1, 2, 4, and 6 [86].

As for most soluble receptors, sTLR2 is generated from the transmembrane TLR2 [86,87]. At least six sTLR2 isoforms have been identified that result from proteolytic cleavage of the full extracellular domain of TLR2 [87]. The different sTLR2s play important roles in the regulation of the immune response to bacterial and viral infections [86]. Similarly to the transmembrane TLR2, sTLR2 binds directly to microbial ligands and, through such a trapping of ligands, the signaling cascade can be diminished, which finally leads to a reduced amount of pro-inflammatory cytokines in the tissues.

Soluble forms of TLR2 have been detected in human fluids such as breast milk, amniotic fluid, saliva [86,87], and in synovial fluid of human joints with osteoarthritis [88]. Intestinal epithelial cells also produce sTLR2. Therefore, it was suggested that through binding of potentially pathogenic molecules in the gut lumen and in body fluids, they may play roles in many human diseases [86]. So far, it is not clear whether sTLR2 exists in homo- or heterodimers. It has been shown that the sTLR2 forms a heterodimer with soluble CD14 in milk and plasma [87]. CD14 is a co-receptor for TLR4, and it is anchored in the cellular membrane [89]. The TLR4/CD14 heterodimer, as already mentioned, binds LPS, while the sTLR4/CD14 heterodimer may recognize additional pathogenic structures.

The soluble forms of TLR4 are produced by other mechanisms (i.e., alternative splicing) other than a post-translational modification. In mice, a shorter mRNA codes for sTLR4, which probably originates by differential splicing [5,6]. Interestingly, the N-terminal end of this sTLR4 consists of a sequence similar to phosphatidylinositol 3-kinase [5], suggesting an activity in kinase pathways. Soluble forms of TLR4 were found in saliva [90] and in synovial fluid of osteoarthritic joints [88]. In addition, sTLR4 has been described as a marker for poor survival of early stage non-small cell lung cancer [91,92]. Whether sTLR4 has a functional role in tumorigenesis remains to be shown.

### 3.5. Non-Bacterial Ligands Interacting with TLRs

An emerging field in nutritional science is the search for dietary compounds from the food chain with the goal to reduce risks and the incidence of gut-related diseases. At least 11 polysaccharides that interact with TLR4 have been identified in eatable plants such as apple or ginseng [93]. In addition to bacterial polysaccharides that either activate or inhibit TLR4, polysaccharides that stimulate TLR4 have been discovered in fungi and algae [93]. Whether such polysaccharides can be used in disease prevention remains to be determined.

TLR-interacting polysaccharides contain mainly α-(1,3), α-(1,4), β-(1,3), and β-(1,4) glyosidic bonds [93]. Of interest is that glucans with β-(1,3) and β-(1,4) linkages, like the ones isolated from oat, barley, and wheat, activate TLR4 [93]. On the other hand, the immune-stimulatory functions of lentinan, the β-glucan of shiitake mushrooms, a β-(1,3)-glucan with β-(1,6) branching, are also well known [94], but the interaction with TLR4 has not been reported yet [93].

β-(2,1)-fructans, to which inulin belongs, are natural carbohydrate storages in many plants [95]. They activate NF-κB in human immune cells by interacting with TLR2, and to a lesser extent with TLR4 and others [93,96]. Thereby, shorter-chain β-(2,1)-fructans induce a more anti-inflammatory cytokine profile compared with long-chain β-(2,1)-fructans [96]. In a cell culture system, β-(2,1)-fructans also protect the integrity of intestinal epithelial monolayers [97]. Pectins, such as lemon pectin, activate TLR2 and TLR4, and increase intestinal epithelial cell barrier function in cellular cultures similarly to β-(2,1)-fructans. The higher the degree of methyl esterification within the pectin, the higher is the activation of the MYD88/NF-κB pathway [98]. In addition, only the lemon pectins with a higher degree of methyl esterification activated TLR2, whereas all tested pectins, even at low doses, activated TLR4 [98].

In addition, natural compounds that regulate the expression of TLRs are of interest to influence inflammation in specific conditions. Paeoniflorin, a monoterpene glucoside, is the principal bioactive constituent of peony root that recently has been associated with a reduced inflammation of the colon in mice models for colitis (dextran sulfate sodium (DSS)-induced colitis) [99]. The mechanism proposed by the authors supports the hypothesis that paeoniflorin decreases the amount of proinflammatory cytokines repression of TLR4 transcription manner [99].

The saturated fatty acids (SFAs) are non-microbial activators of the TLR-signaling pathways [100,101]. Lauric acid (C12:0) activates the TLR4 MYD88-dependent signaling pathway and regulates the expression of several pro-inflammatory genes [100,102,103,104]. A similar effect is also described for the palmitic (C16:0) and stearic (C18:0) acids whose regulation of pro-inflammatory genes occurs primarily via the NF-κB signaling pathway [105,106,107]. Of note, although TLR4 and its co-receptors have been identified as major receptors for SFAs, a clear and direct interaction of TLR4 with these compounds needs to be demonstrated in more detail. Moreover, the activation of other TLRs should not be excluded, as well as the possibility that specific SFAs may activate downstream pathways of TLRs indirectly [104,108,109].

### 3.6. TLR—Microbiota Interaction in IBD

Although TLRs are responsible for detecting and triggering an inflammatory response after recognition of specific pathogenic molecules, they also maintain inflammatory tolerance if the same molecule belongs to commensal bacteria [110,111]. In the GIT, the expression, localization, and distribution of TLRs are directly related to their function of regulating intestinal homeostasis [25,112] that, in part, can be achieved by induction of protective factors stimulated by constitutive detection of ligands belonging to commensal bacteria or by commensal-derived factors induced by damaged epithelium [111].

The relationship existing between TLRs and microbiota is therefore crucial for the onset and progression of specific pathological conditions of the GIT. IBD, for example, is characterized by dysbiosis of the gut microbiota, a general term that defines an abnormal or misbalanced composition of the bacterial ecosystems in the intestinal tract [113,114]. Patients with IBD have an increased abundance of *Enterococcus* spp. and *Bacteroides* spp. accompanied by a decrease of *Bifidobacterium* spp. and *Lactobacillus* spp. levels [115,116,117]. In addition, the IBD is responsible for producing an inflamed intestinal mucosa with a reduced composition in the diversity of the microbiota when compared with a normal tissue [115,118]. Another cellular effect of IBD is, finally, an abnormal compartmentalization of specific TLRs with effects on the inflammation process [25].

In chronic inflammation, the increased expression of TLR2 leads to an exaggerated immune response following NF-κB activation and inflammatory cytokine release [119]. Moreover, TLR2 signaling is critical for the acquisition of tissue-specific functional properties by gut-associated DCs that are also involved in the regulation of other cells of the immune system such as lymphocytes [1,120]. Of note, commensal bacteria such as *B. fragilis* use TLR2 signaling to enhance the colonization of the gut and establish host-microbial tolerance [1,121].

In IBD, the expression levels of TLR4 are also upregulated, and TLR4 signaling has been shown to affect the intestinal microbiota via alterations in gastrointestinal motility that drives clearance of pathogens and maintenance of commensal populations, differentiation of goblet cells, and expression of antimicrobial peptides [1,122,123].

Activation of TLR5 signaling induces differentiation of naive B-cells into plasma cells producing IgA, as well as interleukins (IL-17 and IL-22) promoting early defenses against pathogen invasion of host tissues [1,124].

TLR2, TLR4, and TLR5 have important roles in maintaining intestinal homeostasis. In pathological conditions, the expression and activity of these TLRs is altered, and whether these changes initiate the disease, or whether they represent the effect resulting from pro-inflammatory cytokines release, needs to be verified in more detail [125].

## 4. Outlook

Since TLRs “sense” molecular features specific to pathogen and endogenous threats, it is not surprising that several pathologies develop or progress via activation or deregulation of these receptors.

Elevated expression of TLR2 and 4 was found in the colonic mucosa of children with IBD [70]. Whether these TLRs have a causative role in the development of IBD is currently unknown. Other pathologies associated with deregulation of TLRs activity/expression include diabetes mellitus and rheumatoid arthritis [1,126,127], as well as several pathologies of the central nervous system (CNS). The pathogenicity associated with an altered stimulation of TLRs is, indeed, not only limited to the GIT. TLR4, in particular, is highly expressed in multiple cell types of the CNS such as microglia [128], neurons [129], oligodendrocytes [130], and astrocytes [131], and in the cerebral vascular endothelium [132]. In the brain, TLR4 activation stimulates the up-regulation of pro-inflammatory mediators responsible for driving the neuroinflammation that characterizes pathological conditions such as Alzheimer’s and Parkinson disease [133,134], multiple sclerosis, and amyotrophic lateral sclerosis [135], but also alcoholism, stroke, and traumatic brain injury [136]. These and additional data provide evidence that not only the GIT but also other tissues in the organism can detect peripheral infections or bacterial metabolites and orchestrate an inflammatory response via TLR activation.

TLRs represent the first point of contact between environment and organism, and for this reason altering the activity or the expression of specific TLRs could reduce or prevent the incidence of developing diseases associated with an inflammatory status. To date, polysaccharides are the only compounds identified as non-bacterial ligands of TLR2 and 4 in particular, and therefore they are reported in this manuscript. Identifying novel ligands of TLRs is key to modulating the immune response in the GIT, as well as in other tissues.

## 5. Conclusions

In this minireview we have focused on TLR2, TLR4, and TLR5. We have described their ligands, as well as the signaling cascade events through which these receptors may regulate the inflammatory response in the GIT primarily. Understanding the dynamic equilibrium that exists between microorganisms in the gut and the activity of the immune system regulated by TLRs is a crucial issue for the development and discovery of compounds that are critical to maintaining or re-establishing homeostasis in the GIT, as well as in other tissues of the organism.

## Figures and Tables

**Table 1:** Bacterial ligands of TLR2, TLR4, and TLR5.

TLRs Dimers–Ligand	References
TLR2/1–Triacyl lipopeptides	[40,41,42]
TLR2/1–Heat-labile enterotoxins	[40,43,44]
TLR2/1–Lipomannan/Lipoarabinomannan	[40,45]
TLR2/1–Porins	[40,46,47]
TLR2/6–Diacyl lipopeptides (MALP-2)	[40,41,48]
TLR2/6–Lipoteichoic acid	[40,41]
TLR4/4–LPS (CD14-dependent)	[41]
TLR4/4–LPS (MD-2 dependent)	[41,49]
TLR5/5–Flagellin	[50]

Abbreviations: MALP-2: mycoplasma-derived macrophage activating lipopeptide 2; LPS: lipopolysaccharide; CD14: cluster of differentiation 14; MD-2: known also as lymphocyte antigen 96.
